# Extracting Critical Information from Unstructured Clinicians’ Notes Data to Identify Dementia Severity Using a Rule-Based Approach: Feasibility Study

**DOI:** 10.2196/57926

**Published:** 2024-09-24

**Authors:** Ravi Prakash, Matthew E Dupre, Truls Østbye, Hanzhang Xu

**Affiliations:** 1 Thomas Lord Department of Mechanical Engineering and Materials Science Pratt School of Engineering Duke University Durham, NC United States; 2 Department of Population Health Sciences School of Medicine Duke University Durham, NC United States; 3 Department of Sociology Trinity College of Arts & Sciences Duke University Durham, NC United States; 4 Department of Family Medicine and Community Health School of Medicine Duke Univeristy Durham, NC United States; 5 School of Nursing Duke University Durham, NC United States; 6 Center for the Study of Aging and Human Development Duke University Durham, NC United States; 7 Health Services and Systems Research (HSSR) Duke-NUS Medical School Singapore Singapore

**Keywords:** electronic health record, EHR, electric medical record, EMR, patient record, health record, personal health record, PHR, unstructured data, rule based analysis, artificial intelligence, AI, large language model, LLM, natural language processing, NLP, deep learning, Alzheimer's disease and related dementias, AD, ADRD, Alzheimer's disease, dementia, geriatric syndromes

## Abstract

**Background:**

The severity of Alzheimer disease and related dementias (ADRD) is rarely documented in structured data fields in electronic health records (EHRs). Although this information is important for clinical monitoring and decision-making, it is often undocumented or “hidden” in unstructured text fields and not readily available for clinicians to act upon.

**Objective:**

We aimed to assess the feasibility and potential bias in using keywords and rule-based matching for obtaining information about the severity of ADRD from EHR data.

**Methods:**

We used EHR data from a large academic health care system that included patients with a primary discharge diagnosis of ADRD based on *ICD-9* (*International Classification of Diseases, Ninth Revision*) and *ICD-10* (*International Statistical Classification of Diseases, Tenth Revision*) codes between 2014 and 2019. We first assessed the presence of ADRD severity information and then the severity of ADRD in the EHR. Clinicians’ notes were used to determine the severity of ADRD based on two criteria: (1) scores from the Mini Mental State Examination and Montreal Cognitive Assessment and (2) explicit terms for ADRD severity (eg, “mild dementia” and “advanced Alzheimer disease”). We compiled a list of common ADRD symptoms, cognitive test names, and disease severity terms, refining it iteratively based on previous literature and clinical expertise. Subsequently, we used rule-based matching in Python using standard open-source data analysis libraries to identify the context in which specific words or phrases were mentioned. We estimated the prevalence of documented ADRD severity and assessed the performance of our rule-based algorithm.

**Results:**

We included 9115 eligible patients with over 65,000 notes from the providers. Overall, 22.93% (2090/9115) of patients were documented with mild ADRD, 20.87% (1902/9115) were documented with moderate or severe ADRD, and 56.20% (5123/9115) did not have any documentation of the severity of their ADRD. For the task of determining the presence of any ADRD severity information, our algorithm achieved an accuracy of >95%, specificity of >95%, sensitivity of >90%, and an *F*_1_-score of >83%. For the specific task of identifying the actual severity of ADRD, the algorithm performed well with an accuracy of >91%, specificity of >80%, sensitivity of >88%, and *F*_1_-score of >92%. Comparing patients with mild ADRD to those with more advanced ADRD, the latter group tended to contain older, more likely female, and Black patients, and having received their diagnoses in primary care or in-hospital settings. Relative to patients with undocumented ADRD severity, those with documented ADRD severity had a similar distribution in terms of sex, race, and rural or urban residence.

**Conclusions:**

Our study demonstrates the feasibility of using a rule-based matching algorithm to identify ADRD severity from unstructured EHR report data. However, it is essential to acknowledge potential biases arising from differences in documentation practices across various health care systems.

## Introduction

More than 6 million Americans aged 65 years and older are currently living with Alzheimer disease and related dementias (ADRD), constituting about 11% of the total American population aged 65 years and older [1]. This number is projected to double by 2060, reaching 13.8 million individuals affected by ADRD [1]. Despite the absence of a cure, timely identification of ADRD can significantly improve the quality of life of patients and better prepare their families with essential support resources [2]. Early identification of ADRD will also allow health care professionals and policy makers to develop adequate care programs for both patients and their families. Furthermore, the recent US Food and Drug Administration approval for ADRD treatment, lecanemab-irmb, has demonstrated promising advancements in the pharmacological management of the disease [3]. However, it is important to note that most of these treatments are only targeted at patients with mild cognitive impairment or early-stage dementia. Therefore, identifying ADRD at an early stage of the disease has become even more critical.

Early identification of ADRD poses significant challenges [4,5]. Even among patients with diagnosed ADRD, determining disease severity remains complex from a clinical perspective. Information regarding the presence and severity of ADRD is often limited to structured fields of electronic health records (EHRs) and is more likely to be stored within unstructured EHR sections, such as clinical reports. Moreover, wide variability exists in documentation practices and data structures across different health care systems, specialties, and even among clinicians within the same clinic [6]. Over the past decade, there has been a substantial increase in leveraging information contained in the EHR to improve diagnostic precision [7]. In this context, natural language processing (NLP) has emerged as a promising approach to extract relevant information from EHR data, bridging the gap between structured and unstructured clinical information.

In recent years, EHR data and NLP have been used in various ways to improve ADRD care, such as identifying corelated symptoms [8] and common description of cognitive impairment used by clinicians [9], establishing consensus on cognitive test scores [10], phenotyping of cognitive status [11], and predicting the onset of cognitive decline [12]. However, while structured EHR data have traditionally been used in previous research, they fall short of adequately documenting the severity of ADRD [13]. To address this gap, the unstructured component of EHR becomes critical for capturing essential symptoms and severity indicators related to ADRD [11]. The extent to which unstructured EHR data can be used to determine the severity of ADRD remains largely unknown.

In this study, we assess the feasibility and potential bias of a rule-based matching algorithm for extracting information on ADRD severity in patients with a primary discharge diagnosis of ADRD. Specifically, we develop an algorithm that acquires cognitive test scores and identifies distinct mentions of the presence and severity of ADRD from the primary discharge diagnosis. As each health care system may encode clinical information in the EHR differently, we propose a general framework that health care systems can adopt to tailor their needs and reduce irrelevant “noise” in the EHR—any unwanted irrelevant information.

## Methods

### Data Sources and Study Population

This is a retrospective cohort study that used data from Duke University Health System. Due to the sensitive nature of the data, qualified researchers trained in human subject confidentiality protocols may send requests to access the data that support the findings of this study to the corresponding author.

EHR data were extracted using Duke Enterprise Data Unified Content Explorer, a data extraction system based on Epic (Maestro Care) that identifies patient cohorts and provides access to clinical data stored in the organizational data warehouse [14]. According to previous research, we identified patients with ADRD as those who had at least one clinical encounter at Duke University Health System with at least one principal discharge diagnosis of ADRD based on *ICD-9* (*International Classification of Diseases, Ninth Revision*) or *ICD-10* (*International Statistical Classification of Diseases,* *Tenth Revision*) codes [9,15,16]. The list of *ICD-9/10* codes was based on an established algorithm from previous literature [10,17]. A total of 9115 patients aged 40 years or older, diagnosed with ADRD between January 1, 2014, and December 31, 2019, were included, totaling 65,576 patient records.

### Data Management

As most of the content present in a patient record is not directly related to the severity of ADRD, we used a keyword list that contains common ADRD terminologies to flag patient records and sections of the record that include words directly related to ADRD. The initial list was generated based on previous work [9] and was further modified to include synonyms and additional keywords corresponding to *ICD-9/10* codes related to ADRD. An expert panel of clinicians provided input on the list through an iterative process. The final list (Table S1 in Multimedia Appendix 1) included 38 unique ADRD keywords, consisting of terminologies used by clinicians at Duke University Health System to document ADRD-related information in patient records. This step helped us extract the context in which these words and phrases were mentioned to reduce noise (ie, text not related to the severity of ADRD).

### NLP Algorithms

Unstructured EHR data, characterized by its absence of standardized writing patterns, often manifests inconsistencies in both quality and content. This includes the presence of spelling errors, typographical inaccuracies, formatting inconsistencies, uncommon abbreviations, and other customary challenges inherent in note-taking practices [18]. In response to these challenges, progress in NLP capabilities has helped to filter out important and relevant information from EHR data automatically. This can be done through a rule- or learning-based approach. Previous research has suggested that learning-based methods struggle with interpretability [19] and require a considerable amount of labeled text data, which can hinder scalability. Therefore, in our study, we used a rule-based approach, allowing us to interpret our results clearly and quantify the scope of our algorithm.

Specifically, our algorithm worked by sequentially narrowing down the target tokens (parts of the sentence) to extract information pertaining to ADRD textual mentions (ie, ADRD Text) and cognitive scores (ie, ADRD Cognitive Score). Then the algorithm determined the severity of ADRD based on either the direct mention of keywords pertaining to ADRD severity, or cognitive test scores. We categorized the severity of ADRD into three categories that include (1) mild, (2) moderate-to-severe (ie, advanced stage of ADRD), and (3) no severity indicated. Keywords corresponding to each severity stage are included in Table S2 in Multimedia Appendix 1. In the absence of any direct mention of the severity keywords in patient records, the severity of ADRD was defined based on cognitive test scores (Table S3 in Multimedia Appendix 1). Separate lists of keywords for the severity and cognitive scores were generated to aid the NLP algorithm. For patients whose records contained multiple severity levels from the same source (eg, all from cognitive scores), the more severe ADRD was assigned to the patient. As patients are likely to progress from mild to more advanced dementia over time, the more severe disease information was more likely to be the most updated information. In cases where there were discrepancies between the severity indicated by the explicit keywords and the cognitive scores, we defined the severity of ADRD based on the keywords, as providers may characterize the severity of the condition of the patients comprehensively based on additional assessments besides cognitive tests.

#### ADRD Text

**Figure 1:**
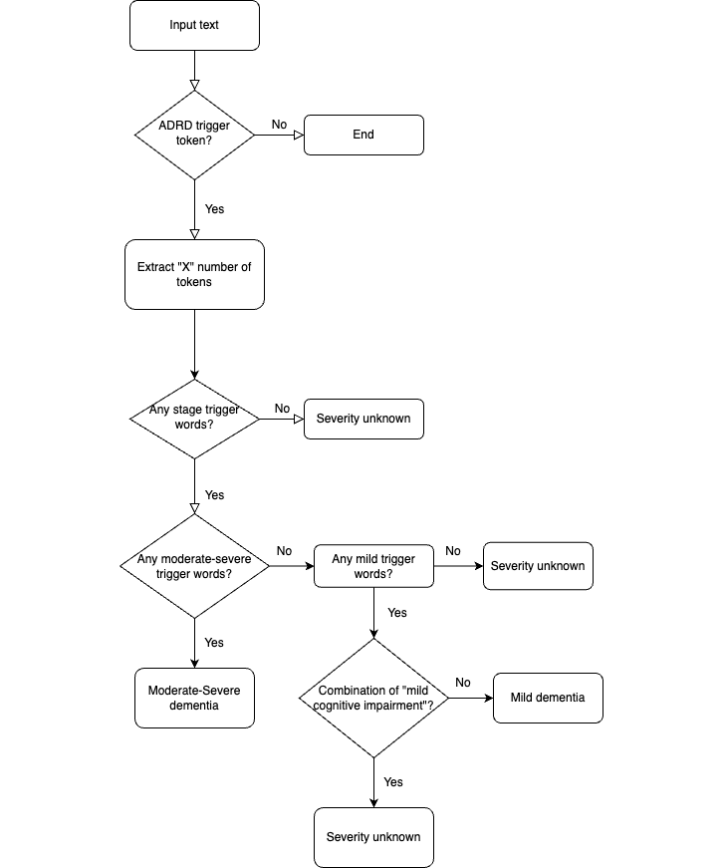
Flowchart to determine the severity of Alzheimer disease and related dementias based on Alzheimer disease and related dementias trigger words. ADRD: Alzheimer disease and related dementias.

We used the Python package spaCy’s pattern-matching function to identify occurrences of words listed in the ADRD keyword list (ie, trigger words). Once a word was identified, we assigned it a positional value of 0. We then extracted 5 tokens before and after the identified word (–5,5), creating a variable with 11 tokens, including the trigger word. The decision to extract this specific number of tokens was based on language constraints and observations from chart reviews, which revealed that most keywords related to the severity of ADRD appeared near the trigger words (eg, “mild dementia”). Next, we performed another pattern search using the severity keywords listed in Table S2 in Multimedia Appendix 1, but this time only on the previously extracted 11 tokens. If a match was confirmed, we extracted the keyword defining the severity of ADRD. The extracted keyword was then assigned a severity category based on the Montreal Cognitive Assessment (MoCA) and Mini Mental State Examination (MMSE) score ranges as outlined in Table S3 in Multimedia Appendix 1. The flowchart for this method is illustrated in Figure 1.

#### ADRD Cognitive Score

**Figure 2:**
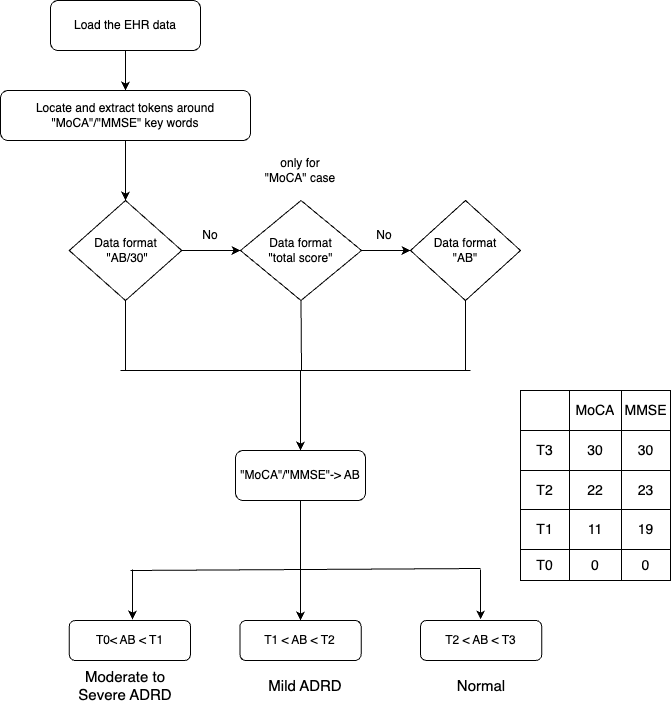
Flowchart to determine Alzheimer disease and related dementias (ADRD) stage based on cognitive test score. EHR: electronic health record; MMSE: Mini Mental State Examination; MoCA: Montreal Cognitive Assessment.

Based on previous literature and input from clinicians, we focused on the cognitive scores from MMSE and MoCA to define the severity of ADRD [16,20]. These 2 cognitive tests were widely used in clinical practice with established cutoffs to determine the severity of cognitive impairment [16,20]. Through chart review and consultation with clinicians, we observed the following patterns for reporting the scores in patient records. For MMSE, the scores were reported either as variations of “AB/30” or “AB”. For MoCA, the scores were reported as variations of “AB/30,” “AB,” or a special case, “score AB,” where A ∈ (0,3), B ∈ (0,9). Some exemplars are present in Table 1. For each patient record, a pattern-matching search was performed to identify occurrences of MoCA and MMSE trigger words. Once a keyword was identified, it was assigned a positional value of 0, and 10 tokens before and after the word were extracted together (–10,10) into a variable. We then used regular expression (Regex) to extract only the numerical score value from the variables with extracted tokens. The above method was used for all the cases mentioned above, with MoCA scores including a special case, due to a slight deviation in terms of extracted tokens, belonging to (0,50) as seen in Figure 2.

**Table 1:** Examples of potential causes of misclassification and representative phrases.

Category and cause of error		Sample sentence	Explanation
**ADRD^a^ text**			
	Incorrect textual representation	Patient demonstrates decreased function secondary to decreased activity tolerance; cognitive deficits; medical status limitations [‘contrast. \r\n\r\n indication: dementia \r\n\r\n findings: \r\n no’, ‘ -- -- -- -- -- -- ’]	Token delimiter missing
	Lack of contextual relationship between texts	Lost 2 points for recalling only 1 out of 3 words after 3 minutes. Her neuropsychological testing concluded that she had evidence of a “mild cognitive disorder, nos.” Mini-cognitive total scoring 1-2 recall and normal cdt: negative for cognitive impairment Vascular dementia with a superimposed severe delirium	Presence of “mild” and “cognitive” words in close vicinity Failure to identify negative context. Presence of “dementia” and “severe” in close vicinity.
**ADRD cognitive score**			
	Particular pattern of reporting MoCA^b^ score	MoCA XX/XX/20XX trails 1 cube 1 clock 2 naming 3 digit span 1 letter a 1 serial 7s 3 sentence repetition 2 fluency 1 abstraction 2 orientation 6 memory 0 education level 0 total score 23	Consistent pattern has been addressed in the algorithm.
	Complex score reporting format	Montreal Cognitive Assessment by on 8/22 (scored 19/30, normal is 26-30/30)	Multiple scores in AB/30 format.
	Human error	MMSE^c^ 36/50	Out of bounds for MMSE score and spelled wrong.

^a^ADRD: Alzheimer disease and related dementias.

^b^MoCA: Montreal Cognitive Assessment

^c^MMSE: Mini Mental State Examination.

#### Sampling, Testing, and Analysis

The rule-based algorithm was fine-tuned on a set of 200 patient records. To test the performance of the algorithm, we generated 2 additional sample files with 200 records each. The index of the first record was chosen using a random number generator to avoid bias. The annotation and validation were done at the level of each patient visit record. A certified clinician reviewed each EHR and determined the severity of ADRD. This manual annotation method served as our gold standard. All discrepancies were reviewed manually by both the data scientist and the clinician and were discussed on a case-by-case basis for resolution. Table 1 was also reviewed by 2 additional team members: 1 data scientist and 1 clinical scientist. We assessed the model performance by comparing the results generated from the algorithm against the gold standard based on the following parameters: *F*_1_-score (ie, the predictive power of the algorithm), accuracy (ie, correct classification), sensitivity, and specificity. In addition, we compared patient characteristics between patients with and without documented dementia severity using Mann‐Whitney U and Pearson chi‐square tests for continuous and categorical variables, respectively. Among patients with documented severity of dementia, we further compared patient characteristics between those with mild ADRD and those with moderate-to-severe ADRD.

### Ethical Considerations

This study was approved by the DUHS Institutional Review Board (Pro00104990).

## Results

A total of 9115 eligible patients were included in the study with over 65,000 records. The median age of the patient population was 78 (IQR 70-84) years. Approximately 60% (5547/9115) of the patients were female, 22.9% (2087/9115) were non-Hispanic Black, and about half were diagnosed by an ADRD specialist (neurologists, neuropsychiatrists, geriatricians, etc). On average, each record contained 931.15 words.

Among all included patients, less than half (3992/9115, 43.8%) had documented information on the stage of their dementia in the EHR. Specifically, about 35% (3190/9115) of the records (3172/9115, 34.8%) included explicit terms that indicated dementia severity, whereas about one-third of the notes (n=2977, 32.7%) included scores from cognitive tests that indicated the severity of patients’ dementia. We found no differences between patients with and without their dementia severity documented with regard to sex (*P*=.45) and race (*P*=.31). However, patients who were older at the time of diagnosis (*P*=.01) and those who were diagnosed at an ADRD specialty clinic or an in-hospital setting (*P*<.001) were more likely to have the severity of their dementia documented in the EHR.

Among those with documented dementia severity, less than 25% (n=920, 23.0%) were determined only based on cognitive scores. In our data, approximately half of these patients (n=1902, 47.7%) were documented to have moderate-to-severe ADRD. Compared with patients with mild ADRD, patients with moderate-to-severe ADRD were more likely to be older.

Table 1 shows potential causes of misclassification and representative phrases from the data sets used. Common causes of these challenges include (1) incorrect textual representation, (2) lack of contextual relationship between texts, (3) either particular or complex patterns of documenting cognitive scores, and (4) human data entry error.

Table 2 presents the performance of our algorithm across 3 sets of data. Overall, the algorithm can identify the presence of information on ADRD severity with high levels of accuracy (*F*_1_-score=0.94, accuracy=0.97, sensitivity=0.94, and specificity=0.98) for training data (set 1). The overall accuracy across the 4 matrices in the 2 testing data sets (sets 2 and 3) was greater than 0.91, except for the *F*_1_-score for set 3. To evaluate the performance of identifying the severity of ADRD, we defined a binary metric with “moderate-to-severe” diagnosis being positive and “mild” being negative. The ability of the algorithm to identify ADRD severity is comparable, if not better than identifying the presence of ADRD severity (*F*_1_-score=0.94, accuracy=0.96, sensitivity=0.88, and specificity=1.0) for training data (set 1). The algorithm had accuracy greater than 0.91 with sensitivity of 1.00 in 2 testing data. The specificity for the 2 testing sets was greater than 0.80.

**Table 2:** Evaluation of ADRD^a^ stage labeling algorithm for patient stage identification and severity of the diagnosis from unstructured EHR data.

Category	Set 1			Set 2			Set 3	
	ADRD information	ADRD severity	ADRD information		ADRD severity	ADRD information		ADRD severity
TP^b^	50	16	21		11	26		15
FP^c^	3	1	3		2	8		0
FN^d^	3	2	1		0	3		0
TN^e^	144	28	171		8	163		11
Accuracy	0.97	0.96	0.98		0.91	0.95		1.0
Sensitivity	0.94	0.88	0.95		1.0	0.90		1.0
Specificity	0.98	1.0	0.98		0.8	0.95		1.0
*F*_1_-score	0.94	0.94	0.91		0.92	0.83		1

^a^ADRD: Alzheimer disease and related dementias.

^b^TP: True positive.

^c^FP: False positive.

^d^FN: False negative.

^e^TN: True negative.

## Discussion

### Principal Findings

In this study, we developed and successfully implemented a rule-based algorithm to identify the severity of ADRD from unstructured EHR data. We detailed the steps to be taken for extracting the relevant information from EHR data and highlighted the challenges associated with it due to heterogeneity in textual representation. We find a lack of access to specialty facilities may impede timely diagnosis and the possibility of treatment at early stages of ADRD progression. As the severity of dementia is critical for health care providers to prescribe appropriate treatment and link resources to patients and their caregivers, our 2-pronged approach to search for relevant information presents a parsimonious yet effective way to make the disease severity information readily available across disciplines and care settings.

Similar to previous research [21-24], we developed the algorithm using a rule-based approach. Starting with an initial list of keywords or phrases based on previous work and contextual clinical knowledge, the list is refined iteratively to identify the target information. This step is followed by sampling the reports from the data set and dividing them into training and testing sets for gold-standard comparison and evaluation. Previous research applied a rule-based approach to identify caregiver availability [21], a record of mild cognitive impairment or Alzheimer disease [22], documentation of cognitive tests [23], and social determinants of health for patients with ADRD [24]. Unlike previous work, where either the rule definition step specific to the medical system [21], included *ICD* (*International Classification of Diseases*) codes [22], or had additional biomarker and cognitive tests information [23]. In comparison, our method shows robustness by using common occurring keywords and points toward the need for defining a minimum number of umbrella rules that have the potential to be generalizable for the entire data set and have better performance. Our developed algorithm is independent of the health care systems and provides clinicians with the flexibility to either use it without any modification or adapt it to their needs. A recent systematic review paper has suggested that rule-based NLP algorithms had similar performance compared with those using more sophisticated methods when the information is scarce in the EHR [25]. In our case, information on the severity of ADRD was presented using a few words, in less than half of the data. To evaluate the performance of our algorithm, we divided the tests into two categories that are (1) identifying the “presence of information on ADRD severity” and (2) “severity of ADRD.” We found our algorithm to be highly accurate in extracting documented information on ADRD severity from the EHR. The performance on sensitivity and specificity also indicates that our algorithm was able to correctly extract stage information where present and reduce false positive results. Taken together, these results support the clinical use of our simplified and generalizable approach to identify the severity of ADRD. Furthermore, compared with previous work, our algorithm showed an improved average *F*_1_-score in identifying the ADRD severity of the condition of the patient [26]. The better performance of our algorithm compared with previous work could be attributed to the use of unstructured EHR data instead of structured EHR and defining clear umbrella rules by identifying recurring patterns in our data set for ADRD severity categorization.

The performance of our algorithm is slightly diminished in identifying the severity of dementia from the records. Upon inspection of the wrongly labeled cases, it can be attributed to (1) the test scores not reflecting the correct severity compared with the clinician’s evaluation, (2) lack of contextual understanding of the sentence, and (3) noise in EHR note (irrelevant information), also noted in previous work [21]. A majority of the abovementioned issues were mitigated by defining subrules [27]. However, any further inclusion would have come at the cost of reduced performance and the need for increased clinician oversight, which limits its generalizability.

Although the rule-based algorithm worked considerably well in most of the cases, it is limited by the patterns and rules defined by the developer. For cases where the token default token delimiter, in our case “space,” is changed or missing, the algorithm fails to extract information. One approach to solve this could be to have an alternative copy of the algorithm that includes other common delimiters (eg, “;” “,” “:”) to identify word tokens. In our evaluation, we only found a few outliers not following the default way of describing texts with “space” as the delimiter. The urge to include all the stray cases would lead to the hard coding of the algorithm and give rise to new challenges with considerable false positive results, making it difficult to comb through. With the development of large language models (LLMs), 1 potential solution might be to use LLM to shape the EHR data in the same format, such as converting all patient records to have equal spacing, removing random commas, etc, without changing textual content and then follow a rule-based approach as presented in this study. Our design philosophy has been to keep the algorithm general while including common patterns. One possible criticism of our study could be dichotomizing the severity of ADRD. The rationale for dichotomizing the severity of ADRD into mild versus moderate-to-severe dementia is related to clinical decision-making. Given that there are several types of ADRD, such as Alzheimer disease, vascular dementia, and Lewy body dementia, to name a few, the differential treatment and care plans are limited as a considerable number of patients have mixed dementia, and the gold standard for a definitive differential diagnosis is still based on autopsy studies. Therefore, for this study, we only focused on a dichotomized version of the severity of ADRD. Future studies should further investigate a more comprehensive classification of ADRD severity and possibly include other tests for diagnosis of ADRD outside MoCA and MMSE.

Overall, we demonstrate the ability of our rule-based algorithm to identify the severity of ADRD, where present, in the EHR and narrow it down to the location of occurrence in the EHR. This not only allows us to comb through valuable unstructured data with ease, but the sequential nature of the algorithm provides us with contextual data that has a high probability of containing information about the severity of dementia. The extracted data can be used in future work to train a machine learning (ML) model with rich and high-quality data. We expect to enhance our method to further identify and predict the progression of ADRD over time. As the performance of an ML model depends on the quality of the data set, following a segmented approach of using a rule-based algorithm for extracting relevant paragraphs from the EHR can be used first to enrich the data set and reduce noise (ie, nonrelevant information from the EHR) followed by model training on the data. Previous research has found that combining structured and unstructured data might be a viable approach to classify patients. With structured data containing useful demographic information and unstructured data containing contextual, patient, and clinical notes, the path forward could be to leverage the qualities of both kinds of data [28] for the use of EHR.

Previous studies that used traditional ML models, such as logistic regression and support vector machine, often include only the structured EHR data due to the limited requirement for data management [6,12,13]. In recent years, increasing numbers of studies have applied deep learning approaches to classify patients for a given condition [6,29,30]. One of the strengths of the deep learning approach is its ability to incorporate relationships between words and a large amount of data in the analysis, which fits the need for using both structured and unstructured data together. Despite its strength, a common criticism against the implementation of deep learning approaches in clinical settings is the lack of interpretability [19]. The use of an interpretable rule-based approach has enabled us to highlight potential biases and pitfalls to be considered when using black-box deep learning models. One potential solution is to use rule-based pattern matching to highlight the trigger words and related neighborhood of words for added context and classify or label the patient record using deep learning techniques [31,32]. With the improvements in LLMs and their enhanced contextual and semantic understanding of texts, our rule-based method can be coupled with a pretrained LLM in pre- or postprocessing of the extracted texts [33]. Care must be taken while using LLMs due to the generative nature of text predictions in avoiding alteration of textual information and being limited to standardizing textual information. The proposed approach needs to be thoroughly evaluated through data privacy and model uncertainty lens before adoption.

We also found that the percentage of patients with missing information on the severity of ADRD is very high. Given the added significance of such information in recent times due to newly approved treatment, it is critical in current clinical practice to improve documentation of the severity of dementia to promote high-quality care. In addition, despite the missing information, our relatively simple algorithm approach has been successful in making previously inaccessible and hard-to-find information readily available to clinicians for a large number of patients. These patients would have otherwise not had this information available to their care team without our very practical approach.

Our study has a few limitations due to the algorithm of our choice and design decisions. First, as mentioned earlier, the rules have to be manually defined and fine-tuned based on a training set. This process, although simple in complexity, can be challenging as EHR can be very different based on health care systems. As the rules are manually defined, it has room for human error. On the other hand, this approach gives the researchers the flexibility to adapt the algorithm structure easily to their health care systems and needs to be fine-tuned. Second, rule-based algorithm matching studies are limited by a lack of contextual understanding between text groups and fail to recognize connotations in sentences. For example, the presence of a negative test result may confuse the algorithm pattern identification process unless explicitly included in the algorithm definition. Third, our approach has been able to only include rules for patterns that are common throughout the data set, such as for the ADRD Cognitive Score function, we include every score defined in the format of AB/30, AB, and a special type of definition as mentioned before. Therefore, we might have missed out on some of the cases straying away from the common patterns. For our instance, attempts to include every unique case led to the results being very irrelevant as it started capturing a lot of unimportant information and the algorithm became very rigid. Fourth, the study determines dementia severity based on cognitive test scores and trigger words for ADRD and does not include medical prescriptions being used by the patient which can be a future direction to make our approach holistic.

Finally, even though we have attempted to keep our method generalizable, the data were extracted from 1 health care system, and the developed rules may not directly apply to data from other health care systems. We have described in detail the steps taken in designing the algorithm with the aim of serving as a baseline approach for research in identifying ADRD severity from unstructured EHR data. The algorithm does the job well for the criteria chosen, and the choice of criteria to include can be a decision of the study designer.

### Conclusion

Rule-based algorithms can provide an interpretable approach to process unstructured EHR data. This study demonstrates the value of unstructured EHR data in providing critical information about ADRD severity from patient records. Pattern-matching rule-based algorithms can be tuned and adapted to health care systems and study-specific needs. The proposed algorithm can serve as a baseline or initial point to shift through pages of EHR reports to identify the most relevant sections or regions. However, it is important to clearly identify the assumptions made, and their limitations while defining the rules. Differences in documentation may also introduce bias in the algorithm as it is fine-tuned. Overall, rule-based algorithms are powerful in handling unstructured EHR data while being transparent and interpretable.

## Appendix

Multimedia Appendix 1 Description of the electronic health record data set.

## Abbreviations

ADRD: Alzheimer disease and related dementias
EHR: electronic health record
*ICD*: *International Classification of Diseases*
*ICD-9*: *International Classification of Diseases, Ninth Revision*
*ICD-10*: *International Statistical Classification of Diseases, Tenth Revision*
LLM: large language model
ML: machine learning
MMSE: Mini Mental State Examination
MoCA: Montreal Cognitive Assessment
NLP: natural language processing
