# Supplementary material for: Extracting Critical Information from Unstructured Clinicians’ Notes Data to Identify Dementia Severity Using a Rule-Based Approach: Feasibility Study
Source: JMIR Aging. 2024 Sep 24;7:e57926. doi: 10.2196/57926 (PMC11462099; doi:10.2196/57926)
Supplement: Multimedia Appendix 1 [file aging_v7i1e57926_app1.doc]

**Original Paper: Extracting Critical Information from Clinicians’ Notes: A Rule-Based Approach to Identify Severity of Dementia from Unstructured Data in Electronic Health Records**

**Ravi Prakash, Matthew E. Dupre**, **Truls Østbye, Hanzhang Xu***

**Duke University, Durham, NC, United States**

**Supplementary:**

1. Alzheimer and Dementia Related Disorder trigger word list.
   1. The words mentioned below are used to narrow down on the region within EHR where ADRD related context has been mentioned.

Table S1. List of ADRD trigger words used to identify sentences with explicit mention of diagnosis related information throughout the dataset.

**ADRD Trigger Word Total Occurrence**

| AD | 4287 |
| --- | --- |
| ADRD | 0 |
| Age-related physical debility | 16 |
| ALZ | 46 |
| Alzheimer | 15533 |
| Alzheimers | 1200 |
| Amnestic disorder | 8 |
| Catatonic disorder due to known physiological condition | 27 |
| Cerebral degeneration | 3 |
| Cognition | 12665 |
| Cognitive | 37857 |
| Cognitive decline | 2837 |
| Cognitive Impairment | 6418 |
| Cognitive state | 46 |
| Cognitive status | 3596 |
| Cognitive function | 561 |
| Cognitive functioning | 189 |
| Degeneration of nervous system due to alcohol | 0 |
| Delirium due to known physiological condition | 44 |
| Dementia | 65698 |
| Dementias | 439 |
| Frontotemporal | 2520 |
| FTD | 492 |
| Lewy body | 3467 |
| MCI | 2605 |
| Neurodegenerative | 926 |
| Neurodegeneration | 68 |
| Neurocognitive Disorder | 6222 |
| Neurocognitive | 7756 |
| Neurocognitive d/o | 93 |
| Other disorders of brain in diseases classified elsewhere | 0 |
| Other persistent mental disorders due to conditions classified elsewhere | 3 |
| Other specified mental disorders due to known physiological condition | 2 |
| Pick’s disease | 13 |
| Senile degeneration of brain | 0 |
| Senility | 6 |
| Systemic atrophy primarily affecting central nervous system in other diseases classified elsewhere | 0 |
| VaD | 3457 |
|  |  |

Figure S 1: Word cloud of trigger words in the document based on frequency of appearance throughout the dataset.


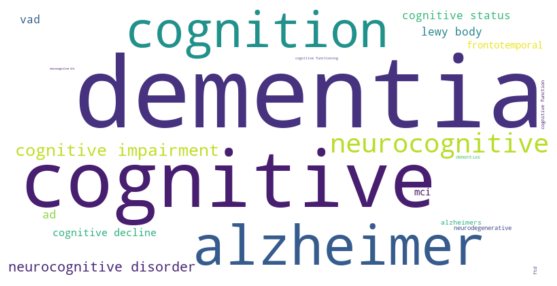


1. ADRD Stage referencing trigger words.
   1. Explicit mention of ADRD stage
   2. Implicit mention of stage through cognitive test score

Table S2. List of keywords present in the vicinity of ADRD trigger words for allocating stage based on, a. textual information, b. extract cognitive test scores.

| Explicit Mention of Stage | Cognitive Test score |
| --- | --- |
| Early  Mild  Early-stage  Mild to moderate  Mild-mod  Mild-moderate  Mild to mod  Moderate  Severe  Advanced  Advance  Late-stage  Moderate to Severe  Mod to Severe  Mod-Severe  Moderate-Severe | MMSE  Mini-mental state examination  Mini-mental state exam  Mini mental state examination  Mini mental state exam  Mini-mental status examination  Mini-mental status exam  Mini mental status examination  Mini mental status exam  MoCA  Montreal Cognitive Assessment |

Table S3. Range of MoCA and MMSE scores used to label ADRD stage based on cognitive test scores

|  | MoCA | MMSE |
| --- | --- | --- |
| Normal | 22-30 | 23-30 |
| Mild ADRD | 11-21 | 19-22 |
| Moderate to Severe ADRD | 0-10 | 0-18 |
